# Correlation of exercise blood pressure levels with concomitant cardiovascular disease

**DOI:** 10.1097/MD.0000000000040226

**Published:** 2024-10-25

**Authors:** Liu Xinwen, Yang Cuicui, Zhou Rongfang, Zhou Jianmei, Ding Fang

**Affiliations:** a The Second Affiliated Hospital of Zhejiang University School of Medicine, Hangzhou, China; b Department of Cardiac Rehabilitation, Zhejiang Hospital, Hangzhou, China; c Zhejiang Cardiovascular and Cerebrovascular Prevention Research Center, Hangzhou, China.

**Keywords:** cardiovascular disease, exercise hypertension, exercise plate test

## Abstract

Hypertension is a fatal but preventable risk factor for cardiovascular disease and an important cause of death from cardiovascular disease. Exercise training has a definite clinical effect on blood pressure control. However, inappropriate exercise is ineffective and may also cause disease. The aim of this study was to evaluate the possible factors influencing blood pressure level in an exercise treadmill test and its relationship with accompanying clinical diseases. Five hundred sixty-four patients who underwent exercise treadmill test were selected and divided into the abnormal exercise blood pressure group (n = 156, age 60.46 ± 9.2 years) and normal exercise blood pressure group (n = 408, age 56.57 ± 8.8 years) according to whether the peak exercise systolic blood pressure was more than or equal to 180 mm Hg. General clinical data and associated clinical diseases were collected from both groups. The prevalence of hypertension and coronary atherosclerotic heart disease in the abnormal exercise blood pressure group was significantly higher than that in the normal exercise blood pressure group (all *P* < .05). At the same time, the smoking rate and glycohemoglobin level of the patients with abnormal exercise blood pressure were significantly increased (all *P* < .05), but there was no statistically significant difference in age, sex, body mass index, low-density lipoprotein cholesterol, high-density lipoprotein cholesterol, and other indicators between the 2 groups (all *P* > .05). Patients with abnormal exercise blood pressure response have a higher prevalence of hypertension and coronary heart disease. Exercise blood pressure level may be an important factor affecting patients’ cardiovascular prognosis.

## 1. Introduction

Hypertension is a fatal but preventable risk factor for cardiovascular disease and an important cause of death from cardiovascular disease. With the increasing aging of the population, the number of patients with hypertension continues to increase, and effective treatment is becoming increasingly urgent.^[1]^ In addition to drug therapy, current evidence^[2–4]^ shows that exercise training has a definite clinical effect on blood pressure control. It can not only optimize the treatment of hypertension but also prevent or delay the progression of hypertension and its complications. However, choosing an appropriate type of exercise is clinically critical. Inappropriate exercise is ineffective and may also cause disease.^[5]^ Exercise hypertension (EH) refers to abnormal hypertension during and/or after exercise, also known as hypertensive response to exercise.^[6]^ It is a manifestation of prehypertension. The ideal blood pressure for exercise should achieve the therapeutic effect of exercise without causing injury to target organs or related clinical diseases.

The innovation of this study lies in its focus on the precise impact of exercise-induced hypertension on cardiovascular outcomes and its efforts to establish safe exercise blood pressure thresholds. Previous studies have demonstrated the benefits of exercise for managing hypertension, but there is a lack of detailed guidelines on what constitutes safe blood pressure levels during exercise to avoid adverse effects. This study aims to fill that gap by analyzing the possible influencing factors of exercise blood pressure levels and examining the relationship between abnormal exercise blood pressure and accompanying clinical diseases. By doing so, it aims to provide a more refined understanding of safe and effective exercise parameters for individuals with or at risk for hypertension. Therefore, this paper discusses the possible influencing factors of exercise blood pressure level and the relationship between abnormal blood pressure and accompanying clinical diseases to provide an important reference for formulating a safe and effective range of exercise blood pressure.

## 2. Data and methods

### 2.1. Clinical data

Study participants were selected from patients who underwent treadmill exercise testing at our hospital. Exclusion criteria included lack of consent, previous participation in the same study, incomplete data, or incomplete exercise tests. A total of 564 patients who underwent exercise treadmill tests in our hospital from January 2017 to January 2019 were selected based on availability and completeness of data. These patients were divided into an abnormal exercise blood pressure group (n = 156, age 60.46 ± 9.2 years) and a normal exercise blood pressure group (n = 408, age 56.57 ± 8.8 years) according to whether the peak exercise systolic blood pressure was greater than or equal to 180 mm Hg.

The sample size was determined based on the retrospective nature of the study, utilizing all available and complete patient data within the specified period. General clinical data, such as sex, age, body mass index (BMI), and smoking history, were collected by 1 experienced observer (Yang Cuicui). Laboratory indicators included total cholesterol (TC), low-density lipoprotein cholesterol (LDL-C), high-density lipoprotein cholesterol (HDL-C), glycohemoglobin (HbA1c), creatinine, and the urinary microalbumin/creatinine ratio. Concomitant clinical diseases such as coronary heart disease, cerebrovascular disease, peripheral atherosclerosis, diabetes, and hypertension were recorded.

This study was approved by the Medical Ethics Committee of Zhejiang Hospital (Ethics Code: ZJH-2021-KC-162), and all participants provided written informed consent.

### 2.2. Inspection methods

#### 2.2.1. Exercise plate test

To standardize the monitoring of blood pressure, we utilized the graded exercise load test of electrocardiograms in this study. This approach allowed for precise measurement of exercise-induced blood pressure.^[6]^ The American Marquette MAX-1 exercise tablet device was used, and the BRUCE protocol was followed for the symptom-limited submaximal exercise test. The target exercise level was set to achieve a maximum heart rate of [(220 ‐ patient age) × (85%–90%)].

#### 2.2.2. Exercise blood pressure measurement

The SUNTECH-tango noninvasive blood pressure monitor is designed to measure upper brachial artery blood pressure throughout various stages of exercise. It can be utilized to track baseline blood pressure during preparation, record blood pressure at 2-minute intervals during each exercise stage, capture peak blood pressure, and monitor blood pressure every 3 minutes during the recovery period (measured in mm Hg).

#### 2.2.3. Coronary angiography

The patient was lying comfortably on a sterile catheter bed. After thoroughly disinfecting the radial artery area and covering it with tissue, the radial artery was punctured using a needle with 1% lidocaine under local anesthesia. A 6F radial sheath was then inserted, followed by the injection of an intrathecal heparin needle. To assess the condition of the coronary arteries, selective coronary angiography was performed, confirming a stenosis of 50% or more in the lumen diameter.

#### 2.2.4. Renal function test

The morning urine sample was analyzed for the microalbumin/creatinine ratio (mg/mmol).

### 2.3. Statistical methods

Epidata 3.0 was utilized for data entry, while SPSS 23.0 was employed for statistical analysis. Quantitative data were presented as mean ± standard deviation, and comparisons between groups were performed using the independent sample *t* test. Frequency and component ratio expressed the count data, with group comparisons conducted via the chi-square test. A *P*-value of <.05 was considered to indicate statistical significance.

## 3. Results

Table 1 compares clinical data between the abnormal exercise blood pressure group (n = 156) and the normal group (n = 408), revealing several key findings. The abnormal group had a higher age average (60.46 ± 9.20 years) compared to the normal group (56.57 ± 8.84 years), with a *t* value of 1.739 and a *P*-value of .112. The BMI was slightly higher in the abnormal group (25.04 ± 2.80 kg/m²) compared to the normal group (24.16 ± 2.87 kg/m²), but this difference was not significant (*t* value = 0.023, *P*-value = .880). The smoke ratio was significantly higher in the abnormal group (41%) compared to the normal group (30%), with a *χ*² value of 6.026 and a *P*-value of .010. HbA1c levels were also significantly higher in the abnormal group (6.72 ± 1.49%) compared to the normal group (6.23 ± 1.20%), with a *t* value of 14.301 and a *P*-value of .000. LDL-C levels were slightly lower in the abnormal group (2.54 ± 1.01 mmol/L) compared to the normal group (2.60 ± 0.92 mmol/L), but this difference was not significant (*t* value = 1.914, *P*-value = .167). Similarly, HDL-C levels were lower in the abnormal group (1.12 ± 0.28 mmol/L) compared to the normal group (1.17 ± 0.29 mmol/L), but this was not statistically significant (*t* value = 0.886, *P*-value = .347). Total cholesterol levels were nearly the same between both groups (4.30 ± 1.24 mmol/L in the abnormal group and 4.33 ± 1.15 mmol/L in the normal group) with no significant difference (*t* value = 1.075, *P*-value = .300). Lastly, urinary microalbumin/creatinine levels were higher in the abnormal group (4.83 ± 14.26 mg/mmol) compared to the normal group (3.71 ± 15.07 mg/mmol), but this difference was not statistically significant (*t* value = 0.183, *P*-value = .669). Compared with the exercise normal blood pressure group, the smoking rate and HbA1c level of the exercise abnormal blood pressure group were significantly increased, with statistical significance (all *P* < .05). There were no statistically significant differences in gender, age, BMI, TC, LDL-C, and HDL-C between the 2 groups (all *P* > .05).

**Table 1:** Comparison of clinical data between exercise blood pressure abnormal group and exercise blood pressure normal group.

Group	N	M [n (%)]	Age (Y)	BMI (kg/m^2^)	Smoke ratio (%)]	HbA1c (%)	LDL-C (mmol/L)	HDL-C (mmol/L)	TC (mmol/L)	Urinary microalbumin/Cr (mg/mmol)
Exercise blood pressure abnormal group	156	117 (75%)	60.46 ± 9.20	25.04 ± 2.80	64 (41%)	6.72 ± 1.49	2.54 ± 1.01	1.12 ± 0.28	4.30 ± 1.24	4.83 ± 14.26
Exercise blood pressure normal group	408	283 (69%)	56.57 ± 8.84	24.16 ± 2.87	123 (30%)	6.23 ± 1.20	2.60 ± 0.92	1.17 ± 0.29	4.33 ± 1.15	3.71 ± 15.07
*t* (*χ*^2^) value		1.739	0.023	0.001	6.026	14.301	1.914	0.886	1.075	0.183
*P* value		.112	.880	.996	.010	.000	.167	.347	.300	.669

BMI = body mass index, Cr = creatinine, HbA1c = glycohemoglobin, HDL-C =** **high-density lipoprotein cholesterol, LDL-C = low-density lipoprotein cholesterol, TC = total cholesterol.

Table 2 shows a comparison of cardiovascular diseases between the abnormal exercise blood pressure group and the normal exercise blood pressure group revealed the following findings. The abnormal exercise blood pressure group (n = 156) exhibited a higher prevalence of carotid/femoral atherosclerosis (52%), cerebrovascular disease (24%), coronary heart disease (40%), coronary atherosclerosis (60%), and hypertension (69%) compared to the normal exercise blood pressure group (n = 408), which showed prevalences of 46%, 25%, 32%, 48%, and 50%, respectively. Statistical analysis indicated significant differences between the 2 groups in the prevalence of coronary heart disease (*χ*^2^ = 3.327, *P* = .043), coronary atherosclerosis (*χ*^2^ = 6.743, *P* = .006), and hypertension (*χ*^2^ = 16.173, *P* = .000). However, no significant differences were found in the prevalence of carotid/femoral atherosclerosis (*χ*^2^ = 1.678, *P* = .115) and cerebrovascular disease (*χ*^2^ = 0.066, *P* = .445). Compared with the normal exercise blood pressure group, the prevalence of hypertension and coronary atherosclerotic heart disease in the abnormal exercise blood pressure group was significantly increased (all *P* < .05). There was no significant difference in the prevalence of cerebrovascular disease and carotid/femoral atherosclerosis between the 2 groups (all *P* > .05).

**Table 2:** Comparison of cardiovascular diseases associated with abnormal exercise blood pressure group and normal exercise blood pressure group.

Group	n	Carotid/femoral atherosclerosis[n (%)]	Cerebrovascular disease[n (%)]	Coronary heart disease[n (%)]	Coronary atherosclerosis [n (%)]	Hypertension[n (%)]
Exercise blood pressure abnormal group	156	81 (52%)	37 (24%)	62 (40%)	94 (60%)	107 (69%)
Exercise blood pressure normal group	408	187 (46%)	101 (25%)	129 (32%)	196 (48%)	203 (50%)
*t* (*χ*^2^) value		1.678	0.066	3.327	6.743	16.173
* P* value		.115	.445	.043	.006	.000

## 4. Discussion

The study compared clinical and cardiovascular data between 2 groups: those with abnormal exercise blood pressure (n = 156) and those with normal exercise blood pressure (n = 408). The abnormal group had a higher average age and BMI, but these differences were not statistically significant. However, the abnormal group had a significantly higher smoking rate (41% vs 30%) and higher HbA1c levels (6.72% vs 6.23%). No significant differences were found in LDL-C, HDL-C, and total cholesterol levels. Additionally, the abnormal group showed a significantly higher prevalence of coronary heart disease (40% vs 32%), coronary atherosclerosis (60% vs 48%), and hypertension (69% vs 50%). There were no significant differences in the prevalence of carotid/femoral atherosclerosis and cerebrovascular disease between the groups. These findings suggest that individuals with abnormal exercise blood pressure are more likely to have higher smoking rates, higher HbA1c levels, and a greater prevalence of certain cardiovascular diseases, highlighting the importance of monitoring and managing these risk factors. According to the China Cardiovascular Health and Disease Report 2019 released in 2020, the mortality rate of cardiovascular diseases ranks first in China, and the estimated number of patients with hypertension in China has reached 245 million. Meanwhile, a report emphasizes that the prevalence rate of hypertension in China is on the rise, and the lack of physical activity is one of the main reasons.^[1]^ In March 2021, the European Association of Preventive Cardiology published a consensus on exercise prescriptions for the prevention and treatment of hypertension. Exercise therapy is believed to be effective in patients with hypertension.^[4]^ Exercise not only delays the progression of hypertension, but also reduces the risk of cardiovascular diseases.^[2–4,7]^ Aerobic exercise can significantly reduce blood pressure and oxidative stress in hypertensive patients,^[8]^ but high-intensity aerobic exercise can lead to an abnormal increase in blood pressure, resulting in endothelial dysfunction and heart damage.^[5]^ At present, the focus of the formulation of exercise intensity is the setting of the target bullseye rate, which often ignores the reasonable control of blood pressure during exercise. Therefore, appropriate exercise intensity and blood pressure monitoring indices should be carefully selected for exercise training.

Exercise hypertension involves multiple factors and systems. According to current research, there are mainly: (1) Abnormal sympathetic and vagus nerve regulation, excessive sympathetic nerve tone during exercise, and vascular autonomic nerve dysfunction in the cardiovascular system, which is characterized by excessive sympathetic nerve activity, are the main participants in hypertension.^[9]^ (2) Overactivation of the renin–angiotensin–aldosterone system and inappropriate exercise overload can lead to excessive secretion of angiotensin II and aldosterone, resulting in enhanced contraction of vascular smooth muscle and sodium retention, leading to an abnormal increase in exercise blood pressure.^[10]^ (3) Abnormal vascular structure and function are more common in the elderly, with vascular sclerosis and decreased arterial elasticity, resulting in increased arterial wall resistance and diastolic function, decreased vascular reserve function, and overreaction to exercise blood pressure.^[11,12]^ In addition, it is worth mentioning that vascular abnormalities represented by endothelial dysfunction, enhanced oxidative stress and vascular remodeling are also considered to be the causes of exercise hypertension.^[13]^ (4) Other factors include genetic, metabolic, and other comprehensive factors.

Because EH is not easy to detect and the current diagnostic criteria are not uniform, it can be easily ignored, which makes EH more harmful. A clinical study published in the Framingham Heart Study with 12 years of follow-up showed that during 12 years of follow-up, 550 people (44.4%) developed new hypertension, 322 people (16.2%) developed first cardiovascular disease, and 300 people (15.1%) died.^[14]^ Higher subextreme exercise blood pressure levels and impaired blood pressure recovery may be markers of late subclinical and clinical cardiovascular diseases, and all-cause mortality during subextreme exercise in middle-aged individuals. Other studies have shown that EH predicts CV events and mortality independently of resting blood pressure and has the strongest predictive value for increased risk during light- to moderate-intensity aerobic exercise.^[15]^ Among the 6578 asymptomatic participants studied by Weiss et al,^[16]^ the survival rate of the blood pressure > 180/90 mm Hg group was also significantly lower than that of the ≤180/90 mm Hg group when a subextreme exercise load was applied in the second stage of the Bruce exercise regimen in the exercise load test (*P* < .001). The risk of cardiovascular death was 1.96 times that of ≤ 180/90 mm Hg, the multivariate adjusted hazard ratio was 1.96, *P* < .001, and the risk of cardiovascular death was 1.08 for every increase of 10 mm Hg in systolic blood pressure (*P* < .05).

Previous studies have shown that EH is closely associated with clinical diseases. (1) EH and hypertension: Studies have found that exercise-induced hypertension in people with significantly normal resting blood pressure may indicate hypertension that cannot be detected by resting blood pressure screening.^[14,17]^ In the Framingham Heart Study,^[14]^ after adjusting for age, sex, blood pressure before exercise, heart rate, BMI, TC, HDL-C, and other factors, higher systolic and diastolic blood pressure during exercise were associated with a higher risk of hypertension. Consistent with previous studies, we found that patients with abnormal exercise blood pressure had a significantly higher prevalence of hypertension than those with normal exercise blood pressure. In conclusion, EH may be an independent risk factor for predicting the onset of hypertension, or an auxiliary indicator for the diagnosis of latent hypertension. (2) Exercise hypertension and diabetes: According to Weiss et al, several large-sample studies reported that among 31,339 subjects, the incidence of EH was approximately 21%–51% in type 2 diabetes.^[6]^ Poor blood sugar control in patients with diabetes can cause oxidative stress, lipid metabolism disorders, vascular endothelial function impairment, and exacerbation of inflammation, leading to vascular sclerosis. This study found that the prevalence of diabetes and HbA1c levels were significantly increased in patients with abnormal exercise blood pressure, suggesting that blood glucose levels may be related to the occurrence of EH. (3) Exercise hypertension and hypertensive target organ damage: Excessive blood pressure response caused by exercise can cause left ventricular hypertrophy, ventricular remodeling, arteriosclerosis, renal function impairment, and damage to other related target organs.^[18,19]^ Our previous studies also showed that arterial elasticity and urinary microalbumin/creatinine values in EH patients were significantly increased,^[20]^ suggesting early hypertensive target organ damage in EH patients. In this study, compared with normal exercise blood pressure, the smoking rate and HbA1c level of patients with abnormal exercise blood pressure, that is, peak systolic blood pressure ≥180 mm Hg during subextreme exercise, were significantly increased. Simultaneously, there was a significant increase in concomitant cardiovascular diseases such as hypertension, coronary heart disease, and coronary atherosclerotic disease. In the future, we should further explore the relationship between exercise blood pressure level and cardiovascular disease and prognosis, conduct long-term follow-up of participants, and conduct a large-sample case-control study to provide a safer and more effective reference for clinical cardiac rehabilitation.

Weaknesses: The study’s sample size is relatively small and limited to a single hospital, which may affect the generalizability of the results. Additionally, the study design is cross-sectional, preventing the determination of causality.

Strengths: This study provides valuable insights into the clinical implications of exercise hypertension and underscores the importance of maintaining optimal exercise blood pressure levels to prevent cardiovascular complications.

Future research directions: Future studies should include larger, multicenter cohorts and employ longitudinal designs to better understand the causal relationships between exercise blood pressure and cardiovascular outcomes. Further research should also explore the mechanisms underlying exercise hypertension and its long-term impact on cardiovascular health.

## 5. Conclusion

In this study, patients with a peak exercise blood pressure ≥180 mm Hg exhibited more clinical complications and a greater risk of related cardiovascular diseases compared to those with normal exercise blood pressure. This finding underscores the significance of monitoring exercise blood pressure levels as an important factor in cardiovascular prognosis. It is recommended that exercise prescriptions be formulated to ensure blood pressure does not exceed 180 mm Hg during exercise. This approach can help reduce the risk of target organ damage associated with exercise hypertension and cardiovascular disease, ultimately contributing to better cardiovascular health outcomes.

## Acknowledgments

We would like to thank the entire medical staff of the Department of Cardiac Rehabilitation, Zhejiang Hospital, Zhejiang Province.

## Author contributions

**Conceptualization:** Liu Xinwen.

**Data curation:** Liu Xinwen, Yang Cuicui, Zhou Jianmei, Ding Fang.

**Funding acquisition:** Liu Xinwen, Ding Fang.

**Investigation:** Yang Cuicui, Zhou Jianmei.

**Resources:** Zhou Jianmei.

**Supervision:** Liu Xinwen, Zhou Rongfang.

**Validation:** Zhou Rongfang, Ding Fang.

**Writing – review & editing:** Zhou Rongfang, Ding Fang.
